# Anodic Voltammetry of Thioacetamide and its Amperometric Determination in Aqueous Media

**DOI:** 10.3390/s8084560

**Published:** 2008-08-04

**Authors:** Dan Cinghiţă, Ciprian Radovan, Daniela Dascălu

**Affiliations:** West University of Timisoara, Laboratory of Electrochemistry, Str. Pestalozzi Nr. 16, 300115, Timisoara, Romania; E-mails: dan.cinghita@cbg.uvt.ro (D.C.); radovan@cbg.uvt.ro (C.R.)

**Keywords:** thioacetamide, cyclic voltammetry, chronoamperometry, boron-doped diamond electrode, anodic oxidation

## Abstract

TAA is a harmful, presumptive pollutant in tap waters and waste waters. Several alternatives have been tested as new possibilities for the anodic determination of TAA in aqueous solutions, simulated waste waters and tap water. The electrochemical behaviour of thioacetamide (TAA) was investigated at a boron-doped diamond (BDD) electrode both in unbuffered 0.1 M Na_2_SO_4_ and buffered solutions as supporting electrolytes. The anodic oxidation of TAA showed well-defined limiting currents or current peaks and a good linearity of the amperometric signal vs. concentration plots. The analytical parameters of sensitivity, RSD and LOD, obtained under various experimental conditions, suggest the suitability of the BDD electrode for electroanalytical purposes. Low fouling effects, good reproducibility and stability, as well as the sharpness of the amperometric signals in both unbuffered/ buffered acidic or neutral media, highly superior to those obtained using a glassy carbon (GC) electrode, recommend the unmodified BDD electrode as a promising potential amperometric sensor for environmental applications, regarding the direct anodic determination of TAA in aqueous media.

## 1. Introduction

Thioacetamide (TAA), also known as thioacetimidic acid, or acetothioamide (CH_3_CSNH_2_), is a widely used sulfur-containing compound both in the laboratory and in various technical applications [1-10] and can also be present in the environment as organic sulfur compounds [1, 4, 5]. TAA is used as a replacement for hydrogen sulfide in qualitative analyses and in hospital practice, as an accelerator in rubber vulcanization, as a reductant additive in the leather, textile and paper industries, generally having a similar role to thiourea [1-3]. The most important recent technical applications and research studies concerning TAA use now, also draw attention to the usefulness of TAA in the preparation of photocatalytic nanocomposites, mesoporous nanomaterials and metal chalcogenide thin films [6-9]. The widespread uses of TAA are unavoidably accompanied by its toxicity and a number of ecotoxic effects in environmental pollution can result from TAA manipulation and its consequent presence in waste waters. These aspects become increasingly important due to high degree of hepatic and neurotoxic injury, and carcinogenity, of TAA, which has been demonstrated under various experimental conditions [10-17]. Thus, in these circumstances the detection of thioacetamide in real time has become more and more important.

The study of the chemical behaviour of TAA, e.g. TAA hydrolysis [18-20], the use of TAA as precipitant reagent, as well as the various methods of quantitative determination by precipitation [21, 22], amperometric or coulometric titration [23-25], redox reactions [26, 27], potentiometry [28], utilization in precipitation and determination of various elements [29-32], anodization of mercury [33], cathodic stripping voltammetry [34, 35], and even determination of TAA and a series of other thioamides by anodic voltammetry at solid metal electrodes [36, 37], and associated problems concerning sulfide and H_2_S electrochemical oxidation or detection [38, 39] have been experimented or applied. The common solid electrodes have rarely been tested for anodic determination of thioamides, with the exception of the application of pulsed amperometric detection (PAD) method [36] and new direct applications, such as determination of organic sulfur-containing compounds [3, 40, 41] and sulfide oxidation [38] at BDD, determination of thiourea at a BDD electrode [3] or thiourea at a copper oxide-copper electrode [2]. So, beside the classical precipitation or potentiometric methods, the electrochemical potentiodynamic variants, for example the use of mercury electrode and cathodic stripping voltammetry, cathodic stripping voltammetry at a silver electrode or pulsed amperometric detection at platinum or gold electrodes, are previous explored alternatives which involve several complications specific for metallic electrode in the presence of thioamidic compounds. Moreover, in the past, relatively little information has been available regarding electrochemical oxidation of simple organic sulphur compounds at solid electrodes [36, 37]. As a result, anodic detection of sulphur organic compounds at solid electrodes has generally not been considered to be viable for quantitative analysis due to the complications caused by inactivation of the electrode surfaces, apart from methods using pulsed amperometric detection (PAD), or pulsed voltammetry study at a platinum rotating disc electrode.

The carbon based electrode materials and especially boron-doped diamond frequently offer suitable conditions for their use as unmodified or modified anodic sensors. The boron-doped diamond is considered an important material for electroanalysis, since it has several valuable electrochemical properties such as a wide potential window in aqueous solution, a low background current, a low sensitivity to fouling, and high chemical and mechanical stability. These characteristics make it significantly superior to other commonly used electrode materials [40-47]. Owing to the new possibilities offered by boron-doped diamond film as an advanced material providing the basis for elaboration of the adequate solid sensors, the electrochemical detection of the sulphur-containing organics is presently a developing subject [3, 40, 41, 46]. The recently reported detection of examples of this type compounds on BDD has stimulated us to explore a more detailed application and to investigate the electrochemical behaviour of TAA and the exploitation of its anodic oxidation characteristics for amperometric determination.

This paper presents the anodic voltammetry and chronoamperometry of TAA in aqueous solutions at a commercial unmodified boron-doped diamond electrode with the aim of establishing an accessible and attractive option in the electrochemical investigation and direct anodic determination of this toxic sulfur-containing compound in water and water solutions systems. To the best of our knowledge, excepting in the context of a symposium a communication of ours, the amperometric detection and determination of TAA at boron-doped diamond electrode in unbuffered or buffered media has not previously been published.

## 2. Experimental Section

Voltammetric and chronoamperometric measurements were carried out in a single compartment three-electrode Metrohm glass cell, specially designed for electroanalytical purposes. The working electrodes were a boron-doped diamond electrode, 3 mm diameter disk electrode or a Metrohm glassy-carbon (GC) 3 mm diameter disk electrode, embedded in the Teflon cylindrical bodies. A Pt foil was used as counter electrode, and a saturated calomel electrode (SCE) as reference electrode. The commercial BDD electrode supplied by Windsor Scientific Ltd. for electroanalytical use, of the same type as in our previous studies [46-48], a mirror-polished doped polycrystalline industrial diamond (microcrystalline; doping degree ∼ 0.1% boron), was previously stabilized in our laboratory by mild electrochemical oxidation and several hundred repeated alternate polarizations by cycling between +1.8 V and -1 V vs. SCE potential limits in a neutral sodium sulfate supporting electrolyte. Before starting each series of electrochemical measurements, the working electrode was cleaned, degreased, simply, on a wetted pad without alumina powder. Only occasionally it was cleaned, after several extended utilizations, with aqueous alumina (0.1 - 0.3 μm) suspension and then carefully washed with a large volume of double-distilled water. A short rest period between measurements combined with brief stirring of the solution without any supplementary cleaning of the electrode between the successive measurements frequently proved a sufficient treatment for continuous operation. The GC electrode tested for a sequential comparison was degreased, polished with aqueous alumina suspension and also carefully washed. Each determination was repeated three times, the good reproducibility of the practically stabilized state of electrode surface being recovered.

The electrochemical device was an Autolab PGstat 20 EcoChemie system controlled by a PC running GPES Software version 4.8. Cyclic voltammetry (CV) in extended and restricted potential range, especially anodic and anodic chronoamperometry (CA) methods were applied with the purpose of evaluating the overall electrode process and the electroanalytical performances. The volume of the explored solution in the cell was large (50 mL). The chronoamperometric response was recorded for electrolysis at controlled potential, in an unstirred solution, selecting a single potential level from the potential range characteristic for the current peak or the limiting current. The time of the current readings was selected for a conventionally quasi-steady state. The quantity of the analyte in the 50 mL solution inside the cell was sufficient and remained practically unchanged throughout the experiment.

The substances were of analytical degree Fluka and Merck reagents. The supporting electrolytes were unbuffered 0.1 M Na_2_SO_4_ (pH 7) and Britton-Robinson buffer (0.04 M for each component) as BR1 (pH 1.96), or a modified Britton-Robinson buffer, as BR2, containing 0.1 M Na_2_SO_4_, (with pH 2.16 or higher by addition of a sodium hydroxide solution), or phosphate buffer, PHB - 0.1 M (pH 7).

The sulfate supporting electrolyte was prepared in two variants, starting from a 0.5 M stock solution, either by dilution with double distilled water or with tap water. The exemplified tests regarding real samples analyzed in a sodium sulfate supporting electrolyte were carried out for a relatively concentrated residual aqueous solution of TAA and for washing water as example of a dilute solution of TAA. In the latter case, the preliminary preparation of the sample for amperometric determination was associated with obtaining the supporting electrolyte by direct dilution of the stock solution with unpurified washing water simultaneously with a low dilution of the analyzed water sample. The voltammograms were recorded at the stationary electrode, in argon purged and de-aerated quiescent solutions, and at room temperature (23±1°C). Concentrations of thioacetamide ranged between 0.005 mM and 0.20 mM.

## 3. Results and Discussion

### a) Voltammetric and chronoamperometric data

**Figure 1.**
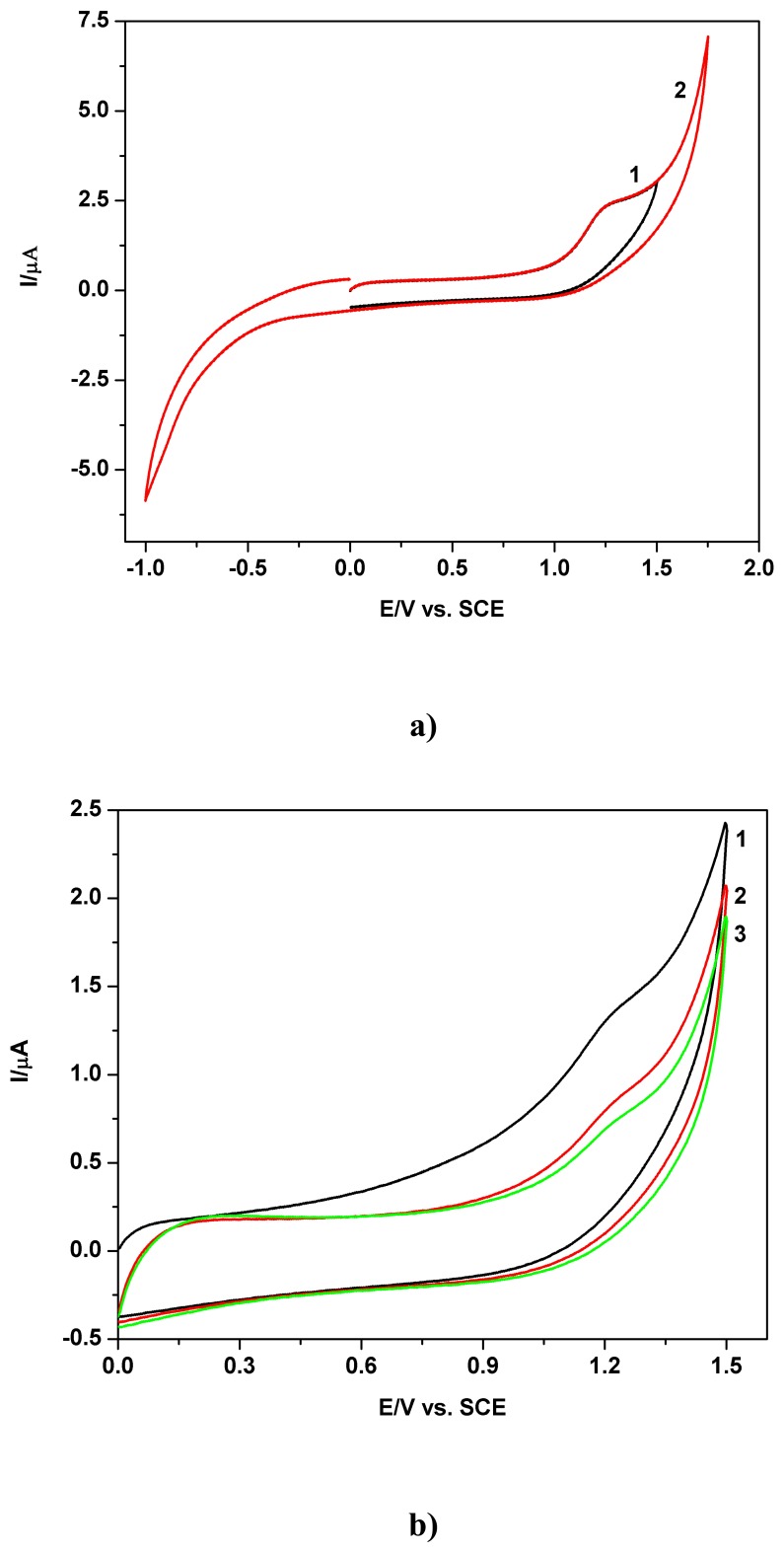
Cyclic voltammograms (CVs) at BDD electrode in presence of TAA and 0.1 M Na_2_SO_4_ pH 7 supporting electrolyte; **a)** Effect of explored potential range on the CVs; 0.02 mM TAA; curve 1 - restricted potential range: 0 V → +1.5V → 0 V vs. SCE; curve 2 - extended potential range: 0 V → +1.8 V → -1 V vs. SCE; first scan - S1; scan rate 0.05 Vs^-1^; **b)** Effect of scan number on the CVs; 0.01 mM TAA; curves 1 -3: scan S1-S3; restricted potential range: 0 V → + 1.5V → 0 V vs. SCE; scan rate 0.05 Vs^-1^.

The typical extended cyclic voltammograms (CVs) obtained at boron-doped diamond electrode in unbuffered sodium sulfate media, starting from 0 V vs. SCE in positive direction, both in extended and relatively restricted potential ranges, are presented in Figure 1a for a 0.02 mM TAA solution and 0.05 Vs^-1^ scan rate. The well defined anodic wave is disposed as a shoulder on the forward branch of cyclic voltammogram curve 1 and recovered on the cyclic voltammogram curve 2, approximately between +1.1 V and +1.4 V vs. SCE. This feature can also be observed in Figure 1b, corresponding to lower TAA concentration and a more restricted positive potential range, where the supplementary scan number effect on the successive CVs can be seen.

**Figure 2.**
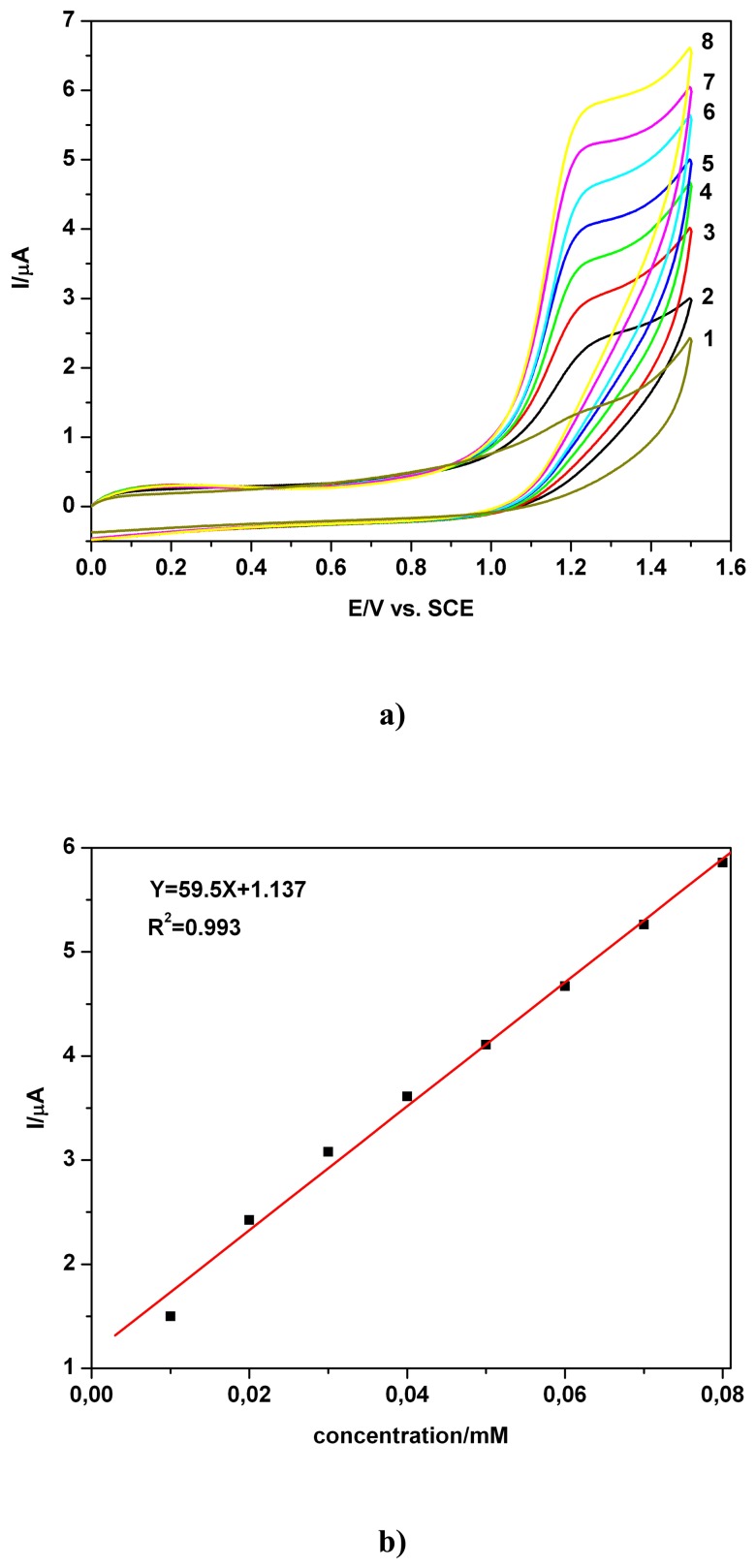
**a)** Cyclic voltammograms (CVs) at BDD electrode in presence of various TAA concentrations and 0.1 M Na_2_SO_4_ pH 7 supporting electrolyte; TAA concentration: 1 - 0.01 mM; 2 - 0.02 mM; 3 - 0.03 mM; 4 - 0.04 mM; 5 - 0.05 mM; 6 -0.06 mM; 7 - 0.07 mM; 8 - 0.08 mM; first scan - S1; scan rate 0.05 Vs^-1^; **b)** corresponding calibration plot I = f (c) at electrode potential around +1.27 V vs. SCE.

The currents (a net peak tendency, not represented here, has been manifested to higher concentration) of TAA oxidation, around +1.27 V vs. SCE, read from Figure 2a (first scan) were a linear function of concentration. The linear calibration plot in the concentration range of 0.01 - 0.08 mM, shown in Figure 2b, was obtained with a very good sensitivity, high correlation parameter and satisfactory LOD (summarized in Table 1), for RSD of 2.5 - 3.1%, is a basis for electroanalytical application (as was certified by several subsequent chronoamperometric data using tap water samples, see further Figures 4b and 4c, which show a high degree of recovery and good linearity).

**Figure 3.**
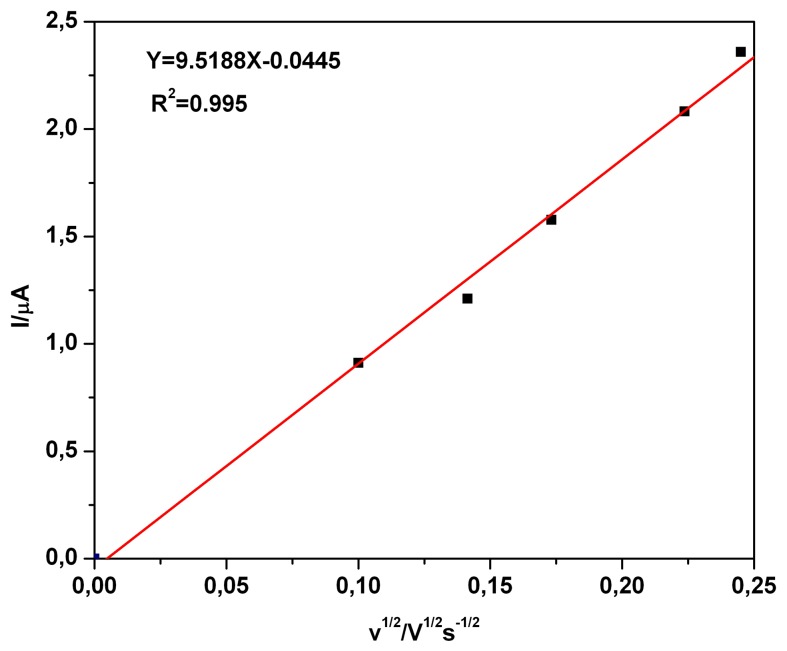
Calibration plot of current vs. square root of scan rate; I = f (v^1/2^); BDD electrode; electrode potential around +1.27 V vs. SCE; 0.02 mM TAA, 0.1 M Na_2_SO_4_ supporting electrolyte pH 7; scan rate: 1 - 0.01 Vs^−1^; 2 - 0.02 Vs^−1^; 3 - 0.03 Vs^−1^; 4 - 0.05 Vs^−1^; 5 - 0.06 Vs^−1^.

The evaluation of the scan rate effect (Figures 3) in the range of 0.01 Vs^−1^ and 0.06 Vs^−1^ on the limiting current was carried out from the CVs obtained at boron-doped diamond electrode for a TAA concentration of 0.02 mM in the restricted potential range. The very good linearity of the current-square root plot, with a practical very low non-zero intercept value in the explored scan rate limits, indicated a diffusely controlled process *and also* that for TAA oxidation in the potential range of limiting current only a weak adsorption process occurred at a BDD.

**Figure 4.**
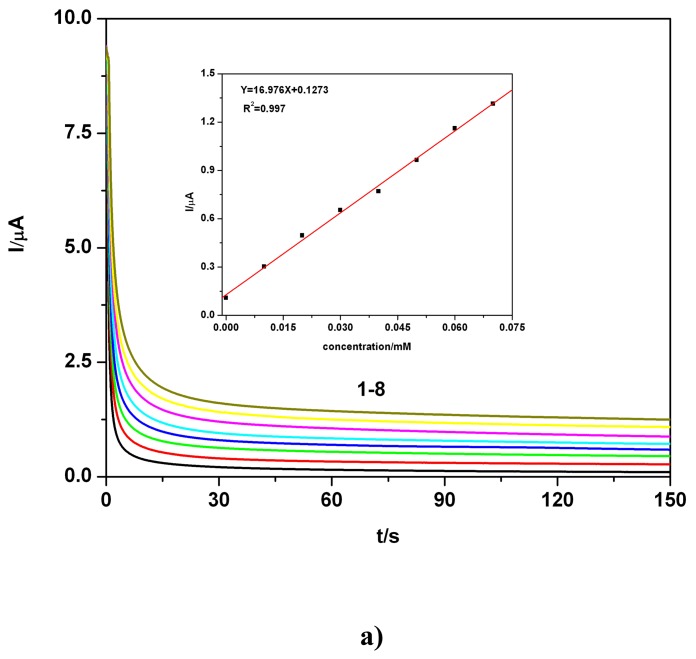

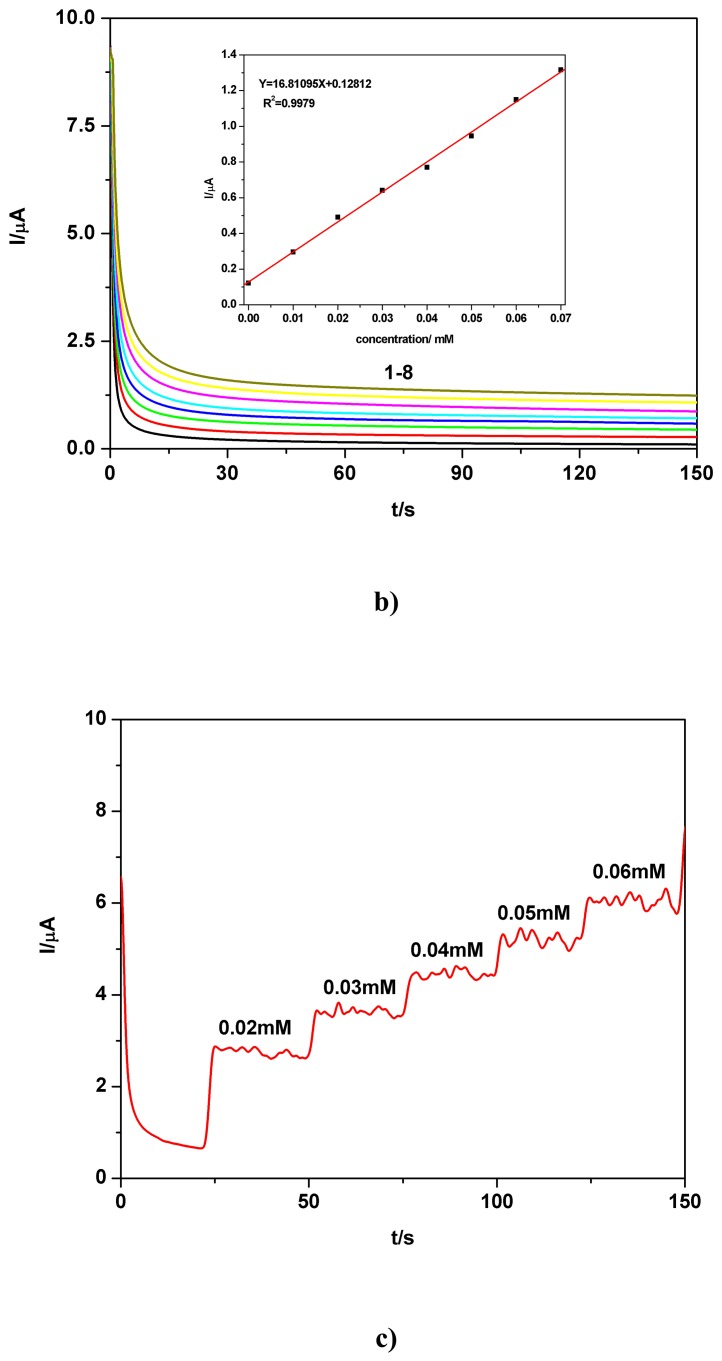

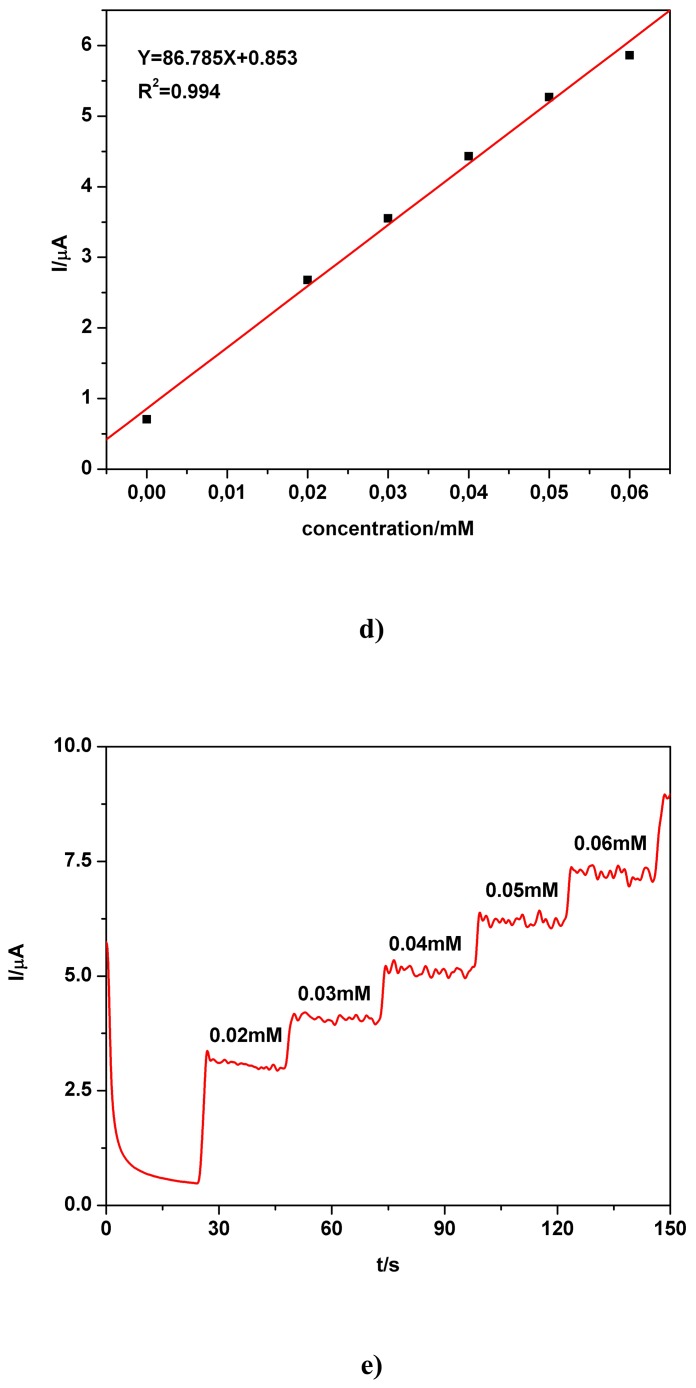

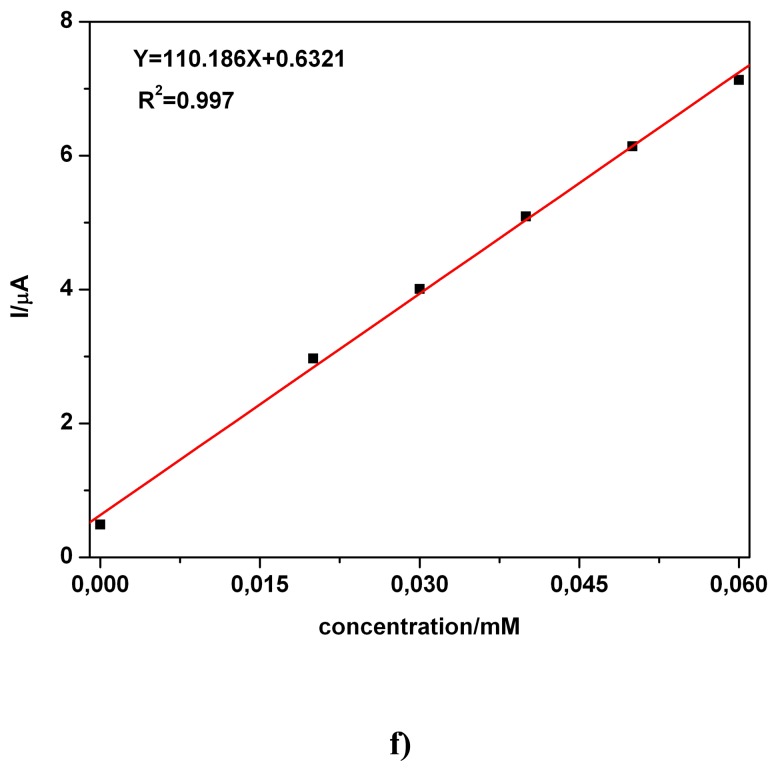
**a)** BDD electrode; Effect of TAA concentration from standard solution prepared in double distilled water on the chronoamperograms (CAs); inset: corresponding calibration plot I = f (c), current readings at 100 s; electrode potential +1.27 V vs. SCE; 0.1 M Na_2_SO_4_ supporting electrolyte pH 7; TAA concentration: 1 - 0; 2 - 0.01 mM; 3 - 0.02 mM; 4 - 0.03 mM; 5 - 0.04 mM; 6 - 0.05 mM; 7 - 0.06 mM; 8 -0.07 mM; **b)** BDD electrode; Effect of TAA concentration from standard solution prepared in tap water on the chronoamperograms (CAs); inset: corresponding calibration plot I = f (c), current readings at 100 s; electrode potential +1.27 V vs. SCE; 0.1 M Na_2_SO_4_ supporting electrolyte pH 7; TAA concentration: 1 - 0; 2 - 0.01 mM; 3 -0.02 mM; 4 - 0.03 mM; 5 - 0.04 mM; 6 - 0.05 mM; 7 - 0.06 mM; 8 - 0.07 mM; **c)** BDD electrode; CA at a stepwise successive addition of TAA, as simulated polluted tap water (“standardized” TAA tap water solution); 0.1 M Na_2_SO_4_ supporting electrolyte pH 7; electrode potential +1.27 V vs. SCE; magnetically stirred solution; without fouling effect; **d)** I = f (c) - calibration plot corresponding to previous condition mentioned in Figure 4b; **e**) BDD electrode; CA at a stepwise successive addition of TAA, standardized TAA and 0.1 M Na_2_SO_4_ supporting electrolyte solutions in double distilled water pH 7; electrode potential +1.27 V vs. SCE; magnetically stirred solution; **f)** I = f (c) - calibration plot corresponding to previous condition mentioned in Figure 4d.

Chronoamperometric data in Figure 4a and the inset showing corresponding linear current-concentration calibration plot with a high degree of the correlation parameter, obtained for the TAA standard solution and the supporting electrolyte in double distilled water, suggest very good possibilities of using chroanoamperometric responses for analytical evaluation. The chronoamperometric data in Figure 4b and the inset showing the corresponding linear current-concentration calibration plot with a high correlation parameter, obtained for the TAA standard solution and the supporting electrolyte in tap water, also suggest the promising option of using chroanoamperometric responses for analytical evaluation, even under apparently less favourable conditions. This aspect has been additionally verified in chronoamperometric experiments using stepwise successive increases of concentration (see also Table 1) achieved by successive addition of TAA standard solution in double distilled water and also by successive stepwise addition of TAA quasi-standard solution in tap water. It can be seen that the results obtained in the two studied situations (successive stepwise addition of TAA “standardized” solution, prepared in tap water media and similar addition of TAA standard solution, prepared in double distilled water exemplified in Figures 4c, 4d, 4e and 4f) show a good agreement between the CA data and the corresponding obtained linear calibration plots.

**Figure 5.**
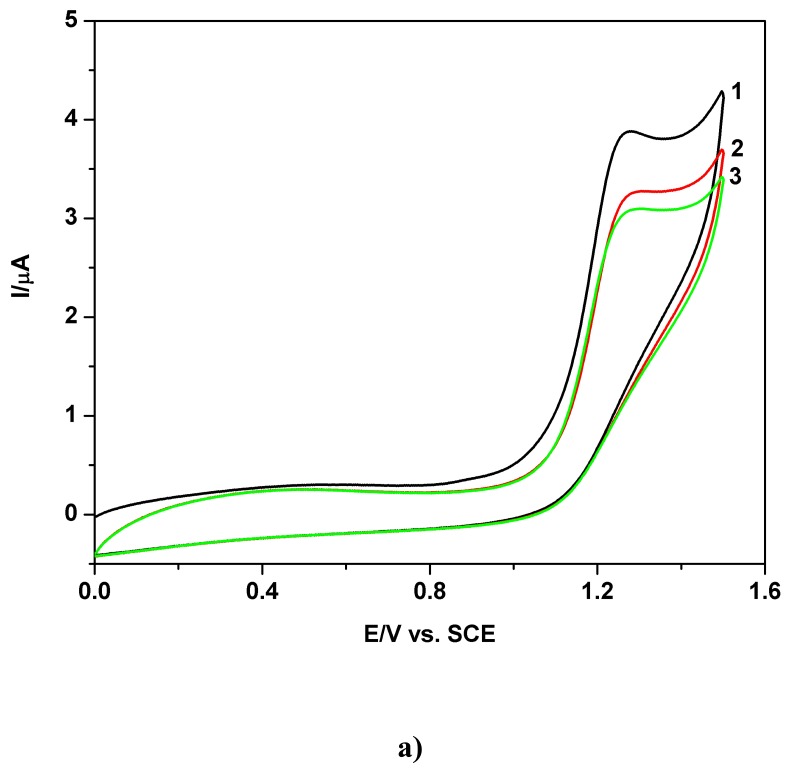

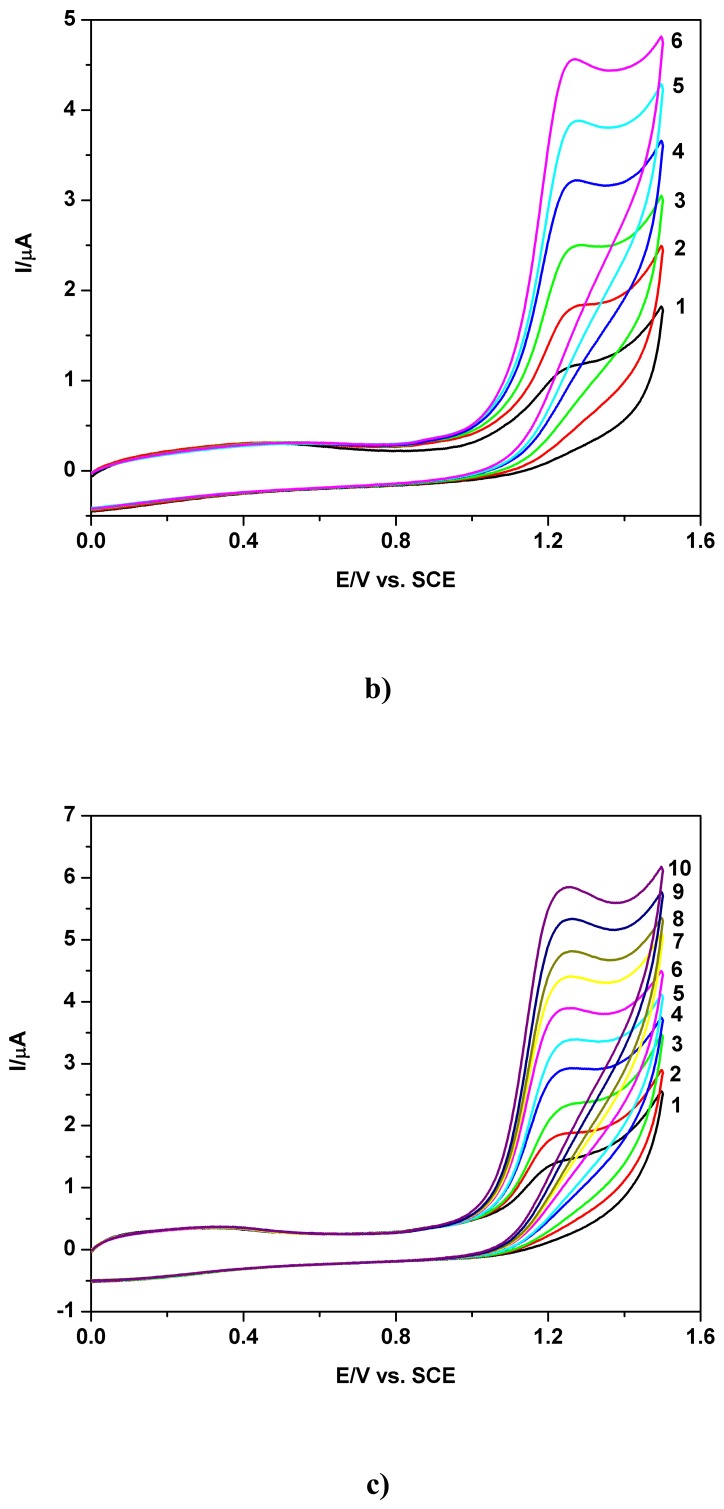

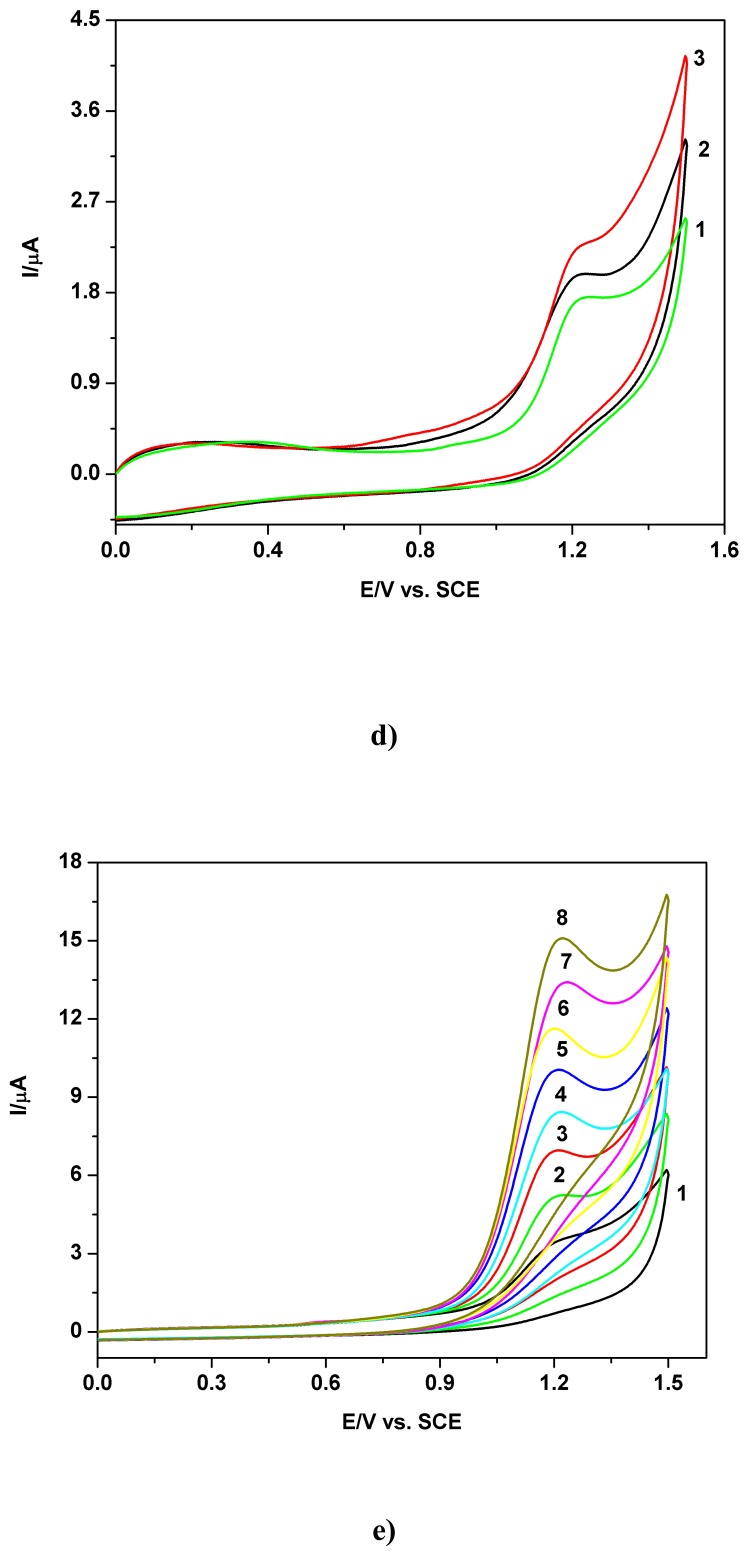

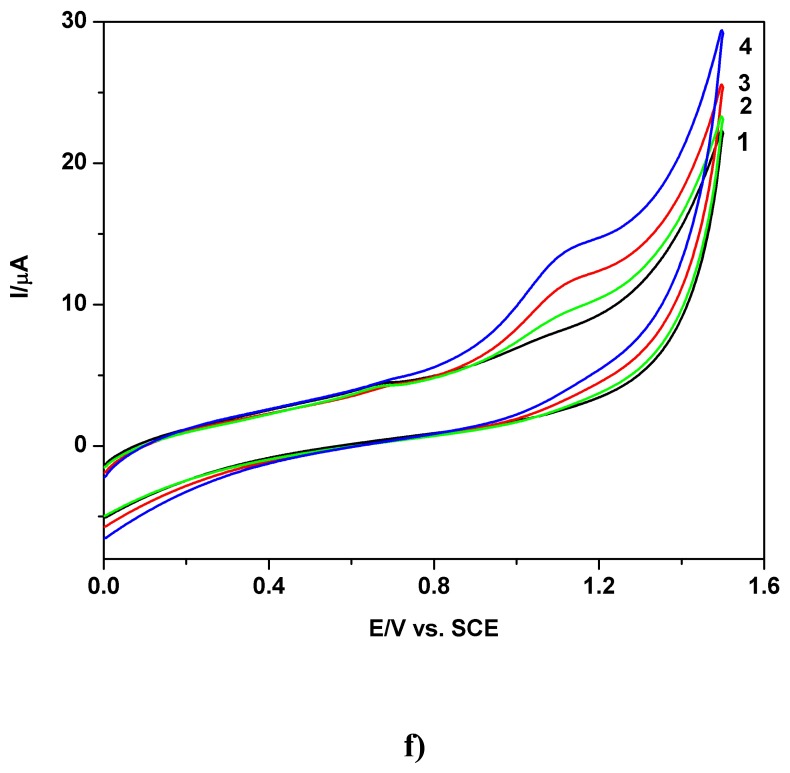
Cyclic voltammograms under various working conditions: **a)** BDD electrode; effect of scan number, 0.05 mM TAA in 0.04 M Britton-Robinson -BR1- buffered supporting electrolyte pH 1.96; 1-3 - scan S1-S3; scan rate 0.05 Vs^-1^; **b)** BDD electrode; effect of TAA concentration; 0.04 M BR1 supporting electrolyte pH 1.96; TAA concentration: 1 - 0.01 mM; 2 - 0.02 mM; 3 - 0.03 mM; 4 - 0.04 mM; 5 - 0.05 mM; 6 -0.06 mM; first scan-S1; scan rate 0.05 Vs-^1^; **c)** BDD electrode; effect of TAA concentration; BR2 supporting electrolyte pH 2.16; TAA concentration: 1 - 0.01 mM; 2 - 0.02 mM; 3 - 0.03 mM; 4 - 0.04 mM; 5 - 0.05 mM; 6 - 0.06 mM; 7 - 0.07 mM; 8 -0.08 mM; 9 - 0.09 mM; 10 - 0.10 mM; first scan - S1; scan rate 0.05 Vs^-1^; **d)** BDD electrode; effect of pH on the CVs; 0.02 mM TAA in BR2; 1 - pH 2.16; 2 - pH 2.86; 3 -pH 4.86; first scan - S1; scan rate 0.05 Vs^-1^; **e)** BDD electrode; effect of TAA concentration on the cyclic voltammograms (CVs); PHB, phosphate buffer, supporting electrolyte pH 7; TAA concentration: 1 - 0.05 mM; 2 - 0.10 mM; 3 - 0.15 mM; 4 - 0.20 mM; 5 - 0.25 mM; 6 - 0.30 mM; 7 - 0.35 mM; 8 - 0.40 mM; first scan - S1; scan rate 0.05 Vs^-1^; **f)** GC electrode; effect of TAA concentration on the cyclic voltammograms (CVs); PHB supporting electrolyte pH 7; TAA concentration: 1 - 0.05 mM; 2 - 0.10 mM; 3 - 0.15; 4 - 0.20 mM; first scan - S1; scan rate 0.05 Vs^-1^.

Similar series of cyclic voltammetry data (Figures 5a to 5f) were obtained at BDD in various buffered media, i.e. in acidic Britton Robinson buffer at two different ionic strength conditions (BR1 and BR2) with a supplementary test to take account of the pH effect, and in neutral phosphate buffer (PHB) when a sequential and supplementary test was made using a GC electrode for the comparison with BDD electrode. A comparative evaluation of electroanalytical data is presented in Table 1.

Successive scans corresponding to the example in Figure 5a showed a well manifested peak current even on the CVs in the second and third scans. The peak currents obtained at 0.05 Vs^-1^scan rate increased progressively and linearly with concentration in all experimental conditions at BDD electrode, as shown in the CV data presented in Figures 5b, 5c, and 5e and the comparative summaries of performance in terms of linearity and calibration parameters in Table 1. Only a small difference in sensitivities was manifested, a very low decrease being observed at a high ionic strength (BR2 supporting electrolyte) which can be attributed to a smaller diffusion coefficient. The low performances of GC suggested by CV data (Figure 5f and Table 1) with high background current and low sensitivity were conditioned by the inferiority of the electrode material. The very flexible and remarkable performances of BDD obtained in various conditions and the absence of a fouling effect are evidence of its overall superiority. The relatively minor effect of pH on the net limiting or peak current value was suggested by the data in Figure 5d. It can also be noted that there is a distortion of voltammograms at alkaline pH (not presented here), probably due to the alkaline conditions superimposing a hydrolysis effect. In the general and comparative context, a neutral sodium sulfate supporting electrolyte is very simple to prepare and offers better conditions for use in TAA detection and determination based on amperometric anodic oxidation data in aqueous media, in standard solutions as well as in simulated contaminated tap water.

### b) Short mechanistic consideration

A related discussion regarding working circumstances for anodic oxidation of TAA in the explored potential range can be considered and considered as follows. Thiocetamide hydrolysis can occur by breakage of two chemical bonds, the carbon-nitrogen and carbon-sulfur, by formation of either thioacetic acid or acetamide intermediates in competitive reactions, but overall hydrolysis [18] can be described by reaction equation:

$${\text{CH}}_{3}{\text{CSNH}}_{2}+2{\text{H}}_{2}\text{O}\to {\text{CH}}_{3}\text{COOH}+{\text{NH}}_{3}+{\text{H}}_{2}\text{S}$$

Thioacetamide solutions are relatively stable in neutral conditions, but thioacetamide will hydrolyse in alkaline media, hydrolysis also being favoured by elevated temperature. In the mild working conditions of our investigation, a relatively good stability of the solutions used has been assumed.

Various successive/simultaneous steps of the oxidation process could be postulated, i.e., a stepwise process [1], with oxidation of sulfur from the oxidation state of −2 to +6, involving the formation of the thioacetamide radical, sulfenyl and sulfenic acid, sulfinic acid, sulfonic acid, or acetamide and even sulfate formation as the concurrent results of C-S bond cleavage, or other variants, in accordance with a general oxidation pathway of sulfur containing compounds [1,3,36,38,40], but the detailed aspects regarding the mechanism were beyond the analytical aim of our paper.

### c) Applicative aspects

**Figure 6.**
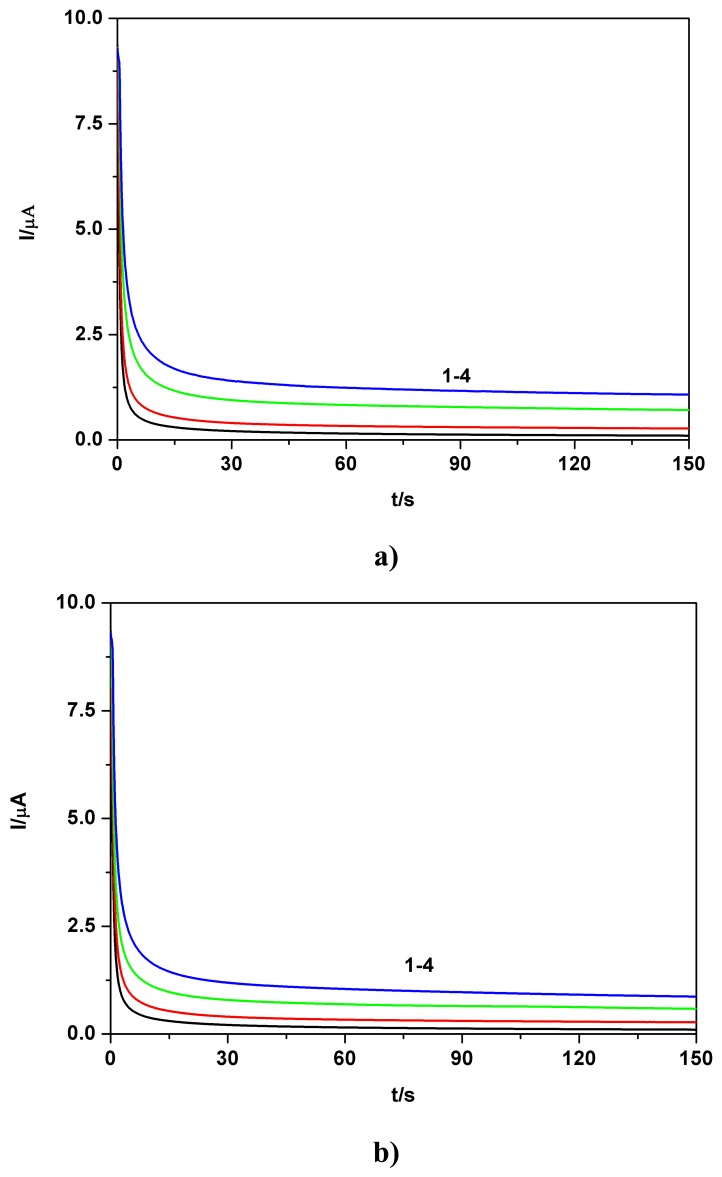
**a)** BDD electrode; Effect of TAA concentration on the chronoamperograms (CAs); current readings at 100 s; electrode potential +1.27 V vs. SCE; 0.1 M Na_2_SO_4_ supporting electrolyte pH 7 (50 mL in the cell); 1 - supporting electrolyte; 2 - addition of 0.01 mM TAA standard solution; 3 - 2 mL of waste water TAA sample and addition of 0.01 mM TAA standard solution; 4 - 2 mL of waste water TAA sample and overall addition of 0.04 mM TAA standard solution; **b)** Effect of TAA concentration on the chronoamperograms (CAs); current readings at 100 s; electrode potential +1.27 V vs. SCE; 0.1 M Na_2_SO_4_ supporting electrolyte and washing water pH 7 (50 mL in the cell); 1 - supporting electrolyte in tap water; 2 - TAA waste water sample (dilution 4/5 with a concentrated sodium sulfate stock solution); 3 - supplementary addition of 0.02 mM standard solution; 4 - 2 mL of waste water TAA sample and overall addition of 0.04 mM TAA standard solution; 5 - supplementary addition of 0.04 mM TAA standard solution; current readings at 100s for analytical evaluation.

The very good calibration parameters, sensitivity and LOD presented in Table 1 confirmed several alternatives for the practical applicability of boron-doped diamond electrode for electroanalytical sensing of TAA. The calibration plot corresponding to progressive TAA addition has been explored in various circumstances as exemplified in Figures 4b and 4c for a tap water “contaminated”, in a simulated fashion, with TAA as a single component pollutant. The neutral unbuffered sodium sulfate supporting electrolyte is very satisfactory in its simplicity and suitability for amperometric measurement using the BDD as working electrode or amperometric sensor. A first complementary example is presented in Figure 6a, which illustrates the application of chronoamperometric evaluation of TA content in a residual relatively concentrated TAA solution as waste water (mixture of a residual stock solution and washing water) from the our student laboratory of analytical chemistry. The evaluation of the concentration of TAA in this real polluted water sample was performed using chronoamperometric data, standard addition method and comparison.

A second example is presented in Figure 6b, showing the application of chronoamperometric evaluation of the TAA content in a washing waste water and the use of the supporting electrolyte in tap water.

The analytical data regarding CAs presented in Figures 6a and 6b correspond to the following concentrations of TAA: 0.0355 M for the residual solution and of 0.0127 mM for the dilute washing waste water.

### d) Additional comments

To the best of our knowledge, the study presented here is the first one published so far on the direct detection of thioacetamide (TAA) in unbuffered and buffered media at a commercial BDD electrode designed especially for as a robust sensor for electroanalytical studies and use. It is true, our main focus was the basics of the method applied to detect TAA directly, in a single step, by anodic oxidation, both voltammetrically and chronoamperometrically. The superiority of such methods over various available alternatives, of measurement, e.g., older “sensitive” methods [34, 35], is obvious, the two major advantages being robustness of the electrode, and the opportunity to carry out the analysis on the field

The significant superiority of BDD electrode as sensor for the anodic amperometric detection and determination of various electrochemical active species is incontestably recognized [40-48], and the general nonspecific sensitivity character of the unmodified electrode is well known. The extended potential windows in the anodic potential range offers favorable conditions for individual detection in single-component systems, and in several more complex situations, allowing even simultaneous detection in the presence of two or several species [43, 44, 47].

Regarding the present paper, the sensitivity can be augmented by hydrodynamic conditions, but interferences from a series of sulfur-containing compounds, such as thiourea [41], sodium diethyldithiocarbamate [46], thiosulfate [49] and others [40], are normal and evident. Their effects in the anodic potential range do not require any additional demonstration.

The simulated and real samples from impure water, explored as a particular example in our investigation, represent a first and very simple stage. We used washing water or exhausted solution resulted in tap water from our analytical chemistry laboratories (TAA is a currently used substitute of the more toxic H_2_S), in direct correlation with our educational activities.

The progressive successive addition in a stirred background solution is a homologated procedure [46, 48, 50]. It is a simple and useful preliminary way which certifies the principal aspects of a continuous detection without an alteration in the linearity of the amperometric signal at a progressive increase in sample concentration. Obviously, in our investigation the progressive successive addition and several BIA experiments were promising chroamperometric alternatives, which precede the application of FIA measurements in our next project.

The sentence “there is no fouling effect” was indirectly certified by linearity of the calibration data obtained by progressive successive addition, as well as by a series of other aspects, as follows: the reproducibility of CVs and the recovery of the amperometric signal by simply stirring of the solution; the very good linearity of the calibration data; the low adsorption effect resulted from the analysis of the scan rate effect; the long life of the electrode with the same voltammetric characteristics; the periodical reproducibility test of the background CVs in neutral supporting electrolyte.

## 4. Conclusions

The voltammetric and chronoamperometric data were obtained at a boron-doped diamond electrode, and sequentially at a glassy carbon electrode, in unbuffered and buffered aqueous media, as a first step in studying the potential application of a solid non-metallic electrode as sensor, suitable for the anodic amperometric assessment of thioacetamide in water solutions or polluted water.

The linear calibration plots showing very good correlation parameters, high sensitivity, good reproducibility and LOD values, recommend the BDD for electroanalytical applications in TAA determination, even in simple and commonly available unbuffered supporting electrolytes such as neutral sodium sulphate.

The absence of electrode fouling and the good linearity of calibration plots obtained from chronoamperometric data, as determined from a stepwise successive addition of TAA, were also confirmed using BDD electrode and TAA standards in deionised water, and TAA in tap water solutions.

The possibility of detecting TAA in single-component aqueous solutions, such as polluted water or waste water, offers a real advantage in situations generated by the use of TAA in the preparation of modern sensitive photocatalytic materials.

The anodic alternative at a boron-doped diamond electrode avoided well-known complications of previously available methods, which involved either cathodic stripping voltammetry or anodic oxidation of TAA and pulsed amperometric detection using a variety of electrocatalytical metal electrodes in alkaline media.

## Figures and Tables

**Table 1.** Electroanalytical parameters of the calibration plot, I *=* aC + b; I (μA), a (μA/mM); C (mM); b (μA); sensitivities, correlation coefficients and LOD values (LOD = 3σ/slope, σ-noise correlated to the minimum explored concentration) for TAA determination in various working conditions.

**Electrode**	**Concentration range (mM)**	Regression equation of linear calibration plot*(μA)	**Sensitivity (μA/mM)**	**R^2^**	**LOD (μM)**	**Supporting electrolyte**	**Method**
BDD	0.02-0.08	I = 59.5C + 1.137	59.5	0.993	1.89	0.1 M Na_2_SO_4_	CV
BDD	0.01-0.07	I = 16.97C + 0.127	16.97	0.997	1.61	0.1 M Na_2_SO_4_	CA_a_**
BDD	0.01-0.07	I=16.8109C+0.1281	16.8109	0.9979	1.585	0.1 M Na_2_SO_4_	CA_b_**
BDD	0.02-0.06	I = 110.18C + 0.632	110.18	0.997	-	0.1 M Na_2_SO_4_	CA^1^
BDD	0.02-0.06	I = 86.785C + 0.853	86.785	0.994	-	0.1 M Na_2_SO_4_	CA^2^
BDD	0.01-0.06	I = 67.97C + 0.4797	67.97	0.999	1.42	BR1 pH 1.96	CV
BDD	0.01-0.06	I = 18.23C + 0.078	18.23	0.995	1.21	BR1 pH 1.96	CA**
BDD	0.01-0.10	I = 49.45C + 0.905	49.45	0.998	2.59	BR2 pH 2.16	CV
BDD	0.005-0.06	I = 14.32C + 0.0016	14.32	0.998	0.84	BR2 pH 2.16	CA**
BDD	0.05-0.40	I = 32.83C + 1.90	32.83	0.999	8.70	PHB pH 7	CV
GC	0.05-0.20	I = 31.38C + 7.48	31.38	0.994	25.81	PHB pH 7	CV

** without background current correction; CA_a_**- TAA standard solution and supporting electrolyte in double distilled water- current readings at 100 s; CA_b_**- TAA standard solution and supporting electrolyte in tap water- current readings at 100 s;

1. stepwise successive addition of TAA standard solution, magnetically stirred solution;

2. stepwise successive addition of tap water containing “standardized” TAA concentration, intensively stirred solution.
